# Effect of visualization of continuous transcutaneous hemoglobin monitoring on iron intake in female university students

**DOI:** 10.20407/fmj.2025-034

**Published:** 2026-09-25

**Authors:** Mikiko Shimizu, Keisuke Iwase, Hironori Tsuzuki, Qiongai Jin, Masato Sugiura, Yumi Akashi, Mariko Umemura, Noriko Suzuki, Tomoko Tanaka, Yukie Kitamura, Yuki Higashimoto, Harumi Kato, Iku Fujiwara, Naoki Yamamoto

**Affiliations:** 1 Faculty of Nursing, Fujita Health University, School of Health Sciences, Toyoake, Aichi, Japan; 2 Department of Nursing, Toyohashi Sozo University, Toyohashi, Aichi, Japan; 3 Department of Nursing Science, Kitasato University School of Health Science, Minamiuonuma, Niigata, Japan; 4 Faculty of Health Care and Nursing, Juntendo University, Urayasu, Chiba, Japan; 5 Department of Nursing, School of Nursing, Tokyo University of Information Sciences, Chiba, Chiba, Japan; 6 Department of Clinical Microbiology, Fujita Health University, School of Medical Sciences, Toyoake, Aichi, Japan; 7 Research Promotion Headquarters, Fujita Health University, Toyoake, Aichi, Japan

**Keywords:** Transcutaneous, Hemoglobin, Anemia, Female university students, Iron

## Abstract

**Objective:**

This study aimed to clarify the impact of visualizing hemoglobin levels through continuous transcutaneous hemoglobin monitoring on iron intake among female university students.

**Methods:**

The study was conducted between December 2022 and February 2023 and involved 11 fourth-year female nursing students from Private University A. Transcutaneous hemoglobin levels were measured at baseline, as well as at 1 month and 2 months post-intervention. The Brief-Type Self-Administered Diet History Questionnaire (BDHQ) was administered at baseline and again at 2 months.

**Results:**

The mean transcutaneous hemoglobin levels (±standard deviation) were 12.8±1.1 g/dL at baseline, 12.5±1.0 g/dL at 1 month, and 13.0±1.0 g/dL at 2 months. A repeated-measures one-way analysis of variance (ANOVA) indicated no significant differences among the measurements. Participants whose transcutaneous hemoglobin levels were below the mean at baseline or at 1 month tended to exhibit an increase in subsequent measurements. The mean change (± standard deviation) from baseline to 2 months demonstrated an increase of 0.3±1.1 g/dL in transcutaneous hemoglobin levels. The change in iron intake was 0.10±1.05 mg/day, with no statistically significant difference observed. A one-way ANOVA revealed no significant differences between grade levels.

**Conclusion:**

For students with low transcutaneous hemoglobin levels, continuous monitoring across three time points—baseline, 1 month, and 2 months—may effectively enhance transcutaneous hemoglobin levels; however, no association with iron intake was identified.

## Introduction

According to the World Health Organization’s survey^1^ on the global prevalence of anemia, approximately 2 billion people worldwide are affected by this condition, including about 30% of women of reproductive age (15–49 years). In Japan, 22.5% of women in this age group are anemic, a rate comparable to that in developing countries, thus constituting a significant public health concern. Anemia has also been highlighted as an issue in Japan’s Fourth Basic Plan for the Promotion of Shokuiku^2^ (food and nutrition education). Additionally, the prevention of anemia has been recognized as a vital component of preconception care,^3^ a growing initiative aimed at promoting health management for women and couples in preparation for future pregnancies.

Furthermore, the prevalence of underweight adults aged 20 years and older, defined by a body mass index (BMI) below 18.5, is reported as 1.7% in the United States (2017–2018^4^) and 1.6%^5^ in the United Kingdom, in stark contrast to 18.4% in Japan (2023).^6^ Evidence suggests that individuals with a BMI below 18.5 are more susceptible to undernutrition and have a higher prevalence of anemia.^7^ Thus, in Japan, where the proportion of underweight individuals often correlates with undernutrition, including anemia, a BMI below 18 has been colloquially termed “Cinderella weight” and acknowledged as a societal issue.

As noted, anemia affects over 20% of Japanese women of reproductive age, making its prevention through preconception care and addressing underweight status a critical public health priority.

As a measure to prevent anemia, the United States legally mandates the production of iron-fortified foods, such as enriched flour, through the Food and Drug Administration^8^ Similar national strategies, including the distribution of supplements, have been implemented in other countries. However, Japan lacks a national policy specifically aimed at anemia prevention, resulting in reliance on municipalities and individuals for preventive efforts. Consequently, this study focused on a transcutaneous hemoglobin measurement device that is accessible for use anytime and anywhere, providing immediate results on-site. The objective of this study was to examine the impact of visualizing hemoglobin levels through continuous transcutaneous hemoglobin monitoring on iron intake among female university students in their 20s and 30s,^6^ who are at the preconception care stage and exhibit a relatively high prevalence of underweight status (BMI <18.5).

## Methods

### 1) Study design

Longitudinal study.

### 2) Definition

In this study, a transcutaneous hemoglobin level of less than 12.0 g/dL was defined as anemia.

### 3) Participants

The participants were fourth-year female nursing students at Private University A who consented to participate in the study.

### 4) Survey period

December 2022 to February 2023.

### 5) Data collection

① Transcutaneous hemoglobin levels were measured at baseline, 1 month, and 2 months using the Rad-67 transcutaneous hemoglobin monitor (Masimo Japan Co., Ltd.). At each time point, three measurements were taken, and the mean value was calculated. The Rad-67 is approved under Japan’s Pharmaceutical Affairs Law. When the dedicated sensor is attached to the fingertip, the transcutaneous hemoglobin level can be confirmed on-site in approximately 1 minute. The Rad-67 employs a multi-wavelength sensor (≥ 7 wavelengths) to differentiate between oxyhemoglobin, deoxyhemoglobin, carboxyhemoglobin, methemoglobin, and plasma. Signal data are collected by passing visible and infrared light (range, 500–1,300 nm) through the capillary bed in the fingertip and measuring changes in absorbance during the blood pulsation cycle. Regarding reliability, data published by Masimo Japan^9^ indicate that the correlation coefficient between transcutaneous hemoglobin values and blood hemoglobin values in 3,519 individuals was r=0.91, with a standard deviation of transcutaneous hemoglobin values of 1.09.

② The Brief-Type Self-Administered Diet History Questionnaire (BDHQ) was distributed and collected at baseline and again at 2 months. This questionnaire is designed for individuals living in Japan and is used to assess, in a relatively straightforward manner, the amount of nutrients habitually consumed from the usual diet, excluding supplements and similar products. In this study, it was utilized to evaluate iron intake. The questionnaire assesses dietary habits over the previous month and consists of one double-sided A3 sheet, with an average completion time of approximately 15 minutes. It is frequently used in large-scale nutritional epidemiologic studies and in individualized health guidance.^10–12^

### 6) Analytical method

BDHQ data were analyzed by a specialized external contractor. Statistical analyses were performed using SPSS version 28.0. To examine changes in transcutaneous hemoglobin levels across measurement time points, a one-way repeated-measures analysis of variance was conducted. The dependent variable was transcutaneous hemoglobin level, and the independent variable was the measurement time point (baseline, 1 month, and 2 months). To evaluate changes in iron intake from baseline to 2 months, a paired t-test was conducted. The correlation coefficient between the changes in transcutaneous hemoglobin levels and changes in iron intake, the correlation coefficient between the change in transcutaneous hemoglobin level and iron intake was calculated to determine their association. The significance level was set at less than 5% for two-tailed tests.

## Ethical considerations

This study received approval from the Fujita Health University Medical Research Ethics Review Committee (Approval No. HM21-582).

## Results

### 1) Participant attributes

Among the 12 fourth-year female nursing students in the Department of Nursing at Private University A who consented to participate in the study, 11 participants with complete data were included in the analysis (mean age, 21.5±0.5 years; mean BMI, 21.1±2.6).

### 2) Longitudinal changes in transcutaneous hemoglobin levels (Figure 1)

The mean transcutaneous hemoglobin level±standard deviation was 12.8±1.1 g/dL at baseline, 12.5±1.0 g/dL at 1 month, and 13.0±1.0 g/dL at 2 months. The repeated-measures one-way analysis of variance showed no significant differences. Among the five participants whose transcutaneous hemoglobin levels increased from baseline to 2 months, three had low baseline transcutaneous hemoglobin levels (two with anemia and one with a level of 12.0 g/dL). Of the 11 participants, four had low baseline transcutaneous hemoglobin levels; two experienced a further decrease at 1 month, followed by an increase at 2 months.

### 3) Changes in iron intake from baseline to 2 months (Table 1)

The mean iron intake was 5.10±1.46 mg/day at baseline and 5.20±1.30 mg/day at 2 months. The paired t-test results showed no significant difference. The number of participants using nutritional supplements increased from three at baseline to five at 2 months (two continued use from baseline and three newly started). One participant who had been using supplements at baseline discontinued use by 2 months. The three participants who newly began using supplements were those whose transcutaneous hemoglobin levels had declined from baseline to 1 month.

### 4) Association between changes in transcutaneous hemoglobin levels and iron intake from baseline to 2 months (Figure 2, Table 1)

The mean change in transcutaneous hemoglobin level was 0.3±1.1 g/dL, and the mean change in iron intake was 0.10±1.05 mg/day. The Spearman rank correlation coefficient between changes in transcutaneous hemoglobin levels and changes in iron intake was r=–0.036, indicating no significant association. All four participants who were anemic at 1 month demonstrated an increase in transcutaneous hemoglobin levels by 2 months.

## Discussion

The novelty of this study lies in its investigation of the impact of continuous transcutaneous hemoglobin monitoring on the prevention and enhancement of anemia.

No significant changes were observed in the longitudinal transcutaneous hemoglobin levels. The limited sample size of 11 participants and the variability in the data might have contributed to the absence of a significant difference. However, a comparison of baseline and 2-month values revealed an upward trend. Participants who exhibited increased transcutaneous hemoglobin levels at the 2-month mark were those with low baseline levels. Participants with low transcutaneous hemoglobin levels at 1 month showed improvements by the 2-month assessment. These findings suggest that participants who received low transcutaneous hemoglobin results, or those whose levels had declined from the previous measurement, might have taken proactive steps to enhance their condition. Specifically, among the eight participants who were not using nutritional supplements at baseline, three had commenced use by the 2-month assessment, all of whom were individuals whose transcutaneous hemoglobin levels had decreased from baseline to 1 month. This indicates that a decline in transcutaneous hemoglobin levels might have prompted the behavior of initiating nutritional supplement use.

Similar to previous studies^13–15^ demonstrating that visualizing numerical indicators, such as blood pressure, body weight, and smartphone usage time, effectively reduces those respective metrics, the present findings suggest that the visualization of values through transcutaneous hemoglobin measurement might have had a comparable effect. Conversely, the BDHQ is a scale that evaluates dietary intake over the preceding month; if it were readministered at 1 month, the assessment period would overlap with that of the baseline survey, potentially hindering the reflection of dietary habit changes. Therefore, this study assessed dietary habits at only two time points (baseline and 2 months) to capture changes in dietary behavior. Consequently, since the timing of nutritional supplement use was evaluated solely at baseline and 2 months, it remains unclear whether the initiation of new supplement use was a direct response to the decline in transcutaneous hemoglobin levels at 1 month or whether it began sometime after the 1-month measurement. This represents a limitation of the current study, and future studies should seek to obtain more detailed information regarding the timing of nutritional supplement use.

Among participants with low baseline transcutaneous hemoglobin levels, the longitudinal patterns exhibited variability: some participants demonstrated gradual increases at both 1 month and 2 months, while others showed a decrease at 1 month followed by an increase at 2 months. This variation suggests that some participants might have commenced coping behaviors after receiving a low baseline result, whereas others might have initiated such behaviors after receiving two consecutive low results, either at baseline and 1 month or after a decline from baseline to 1 month. These findings suggest that to foster coping behaviors aimed at improvement among participants with low transcutaneous hemoglobin levels, it is essential to measure transcutaneous hemoglobin at least twice: at baseline and again at 1 month. Furthermore, establishing a remeasurement at 2 months to assess the effects of coping behaviors implies that conducting a minimum of three transcutaneous hemoglobin measurements may be beneficial for improving transcutaneous hemoglobin levels.

In this study, changes in dietary habits were utilized as indicators of coping behavior intended to improve transcutaneous hemoglobin levels. The BDHQ dietary history questionnaire was administered at baseline and 2 months. A paired t-test of iron intake at baseline and 2 months showed no significant difference. In addition, there was no correlation between the changes in transcutaneous hemoglobin levels and iron intake from baseline to 2 months. Although the small sample size may have contributed to the absence of significant differences or correlations, no association was identified between transcutaneous hemoglobin levels and iron intake in this study. The BDHQ is a dietary history tool,^16,17^ and the actual extent of iron absorption within the body remains unknown. Therefore, differences in iron absorption due to food combinations might have influenced the results. In addition, although the BDHQ includes questions regarding the frequency of nutritional supplement use, it does not inquire about the types of supplements taken. In recent years, a diverse array of nutritional supplements has emerged, and even among iron supplements, there is considerable variation in iron content, type (heme iron or non-heme iron), and the frequency of daily doses. Therefore, the effects of nutritional supplements might also have influenced the findings. Moreover, iron loss due to menstruation among the participants might have further affected the results.

## Limitations of the study

This study is subject to three primary limitations. First, the sample size was small, comprising only 11 participants, which might have resulted in insufficient statistical power. Second, a more comprehensive analysis of dietary habits, involving not only the BDHQ but also an investigation of factors such as the timing of nutritional supplement initiation, the types of supplements used, daily intake frequency, and food combinations, would have been beneficial. Third, analyses that account for menstrual iron loss were necessary.

## Conclusion

Among students with low transcutaneous hemoglobin levels, continuous monitoring at three time points—baseline, 1 month, and 2 months—may effectively improve these levels. However, no association was identified between increases in transcutaneous hemoglobin levels and iron intake. Therefore, future studies should include comprehensive dietary assessments that account for factors such as iron absorption and food combinations, as well as evaluations that consider the menstrual cycle.

## Figures and Tables

**Figure 1:**
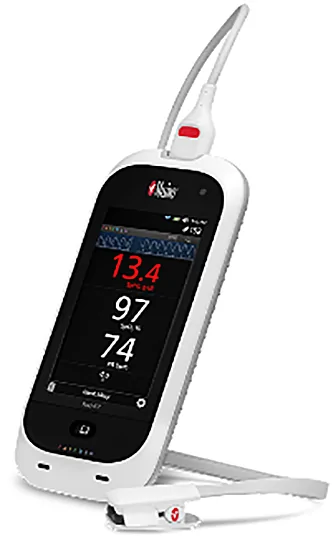
Rad67 (Masimo Co.)

**Figure 2:**
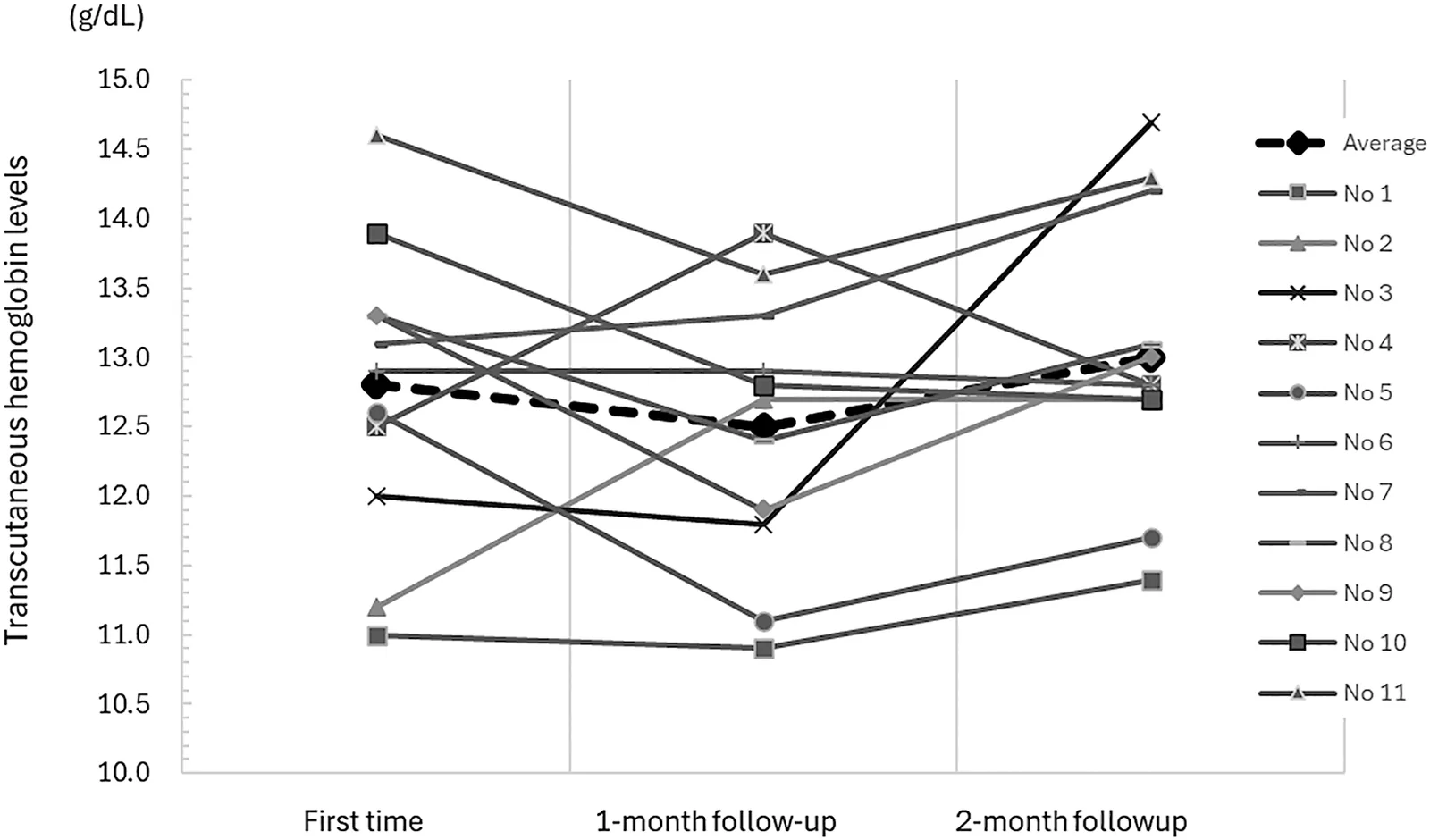
Change in transcutaneous hemoglobin levels from the first time to 2 months (N=11)

**Table 1:** Change in daily Fe intake from the first time to 2 months

	First time		2-month follow-up		Amount of change between the first time and 2-month follow-up
	Daily Fe intake (mg/day)	Use of supplements	Daily Fe intake (mg/day)	Use of supplements	
Average±SD	5.10±1.46		5.20±1.30		0.10±1.05
No. 1	6.73	No	6.90	No	0.17
No. 2	4.74	**Yes**	4.71	**Yes**	–0.02
No. 3	4.50	No	4.11	No	–0.39
No. 4	4.43	No	5.91	No	1.48
No. 5	7.05	No	4.61	Yes	–2.44
No. 6	7.33	No	7.75	No	0.42
No. 7	4.76	No	4.89	No	0.12
No. 8	3.82	Yes	3.67	Yes	–0.15
No. 9	9.84	**Yes**	7.32	**No**	–2.52
No. 10	5.40	**No**	6.09	**Yes**	0.69
No. 11	2.46	No	3.93	Yes	1.47

* No. 1～11: First transcutaneous hemoglobin levels in order of lowest to highest Corresponding to the numbers in Figure 2

## Supplementary Material

PDF-Japanese
