# Supplementary material for: Effect of visualization of continuous transcutaneous hemoglobin monitoring on iron intake in female university students
Source: Fujita Med J. 2026 Sep 25;12(4):312–6. doi: 10.20407/fmj.2025-034 (PMC13724194; doi:10.20407/fmj.2025-034)
Supplement: Supplementary file 1 [file fmj-12-312_s1.pdf]

タイトル：

女子大学生を対象とした継続的な経皮的ヘモグロビン測定値の可視化が鉄摂取量に与える影響の検討

Effect of Visualization of Continuous Transcutaneous Hemoglobin Monitoring on Iron Intake in Female University Students

ランニングタイトル：経皮的ヘモグロビン値の継続的測定の効果
The Effects of Continuous Transcutaneous Hemoglobin Monitoring

著者：

Mikiko Shimizu, RN, MW, PHN, PhD [1], Keisuke Iwase, RN, MS [1], Hironori Tsuzuki, RN, MS [2], Qiongai Jin, RN, PhD [1], Masato Sugiura, RN, MS [1], Yumi Akashi, MS [1], Mariko Umemura, RN, MS [1], Noriko Suzuki, RN, PhD [3], Tomoko Tanaka, RN, PhD [4], Yukie Kitamura, RN, PhD [5], Yuki Higashimoto, PhD [6], Harumi Kato, RN, PhD [1], Iku Fujiwara, RN, MS [1], Naoki Yamamoto, PhD [7]

[1] Faculty of Nursing, Fujita Health University, School of Health Sciences, Toyoake, Aichi, Japan
[2] Department of Nursing, Toyohashi Sozo University, Toyohashi, Aichi, Japan
[3] Department of Nursing Science, Kitasato University School of Health Science, Minamiuonuma, Niigata, Japan
[4] Faculty of Health Care and Nursing, Juntendo University, Urayasu, Chiba, Japan
[5] Department of Nursing, School of Nursing, Tokyo University of Information Sciences, Chiba, Chiba, Japan
[6] Department of Clinical Microbiology, Fujita Health University School of Medical Sciences, Toyoake, Aichi, Japan
[7] Research Promotion Headquarters, Fujita Health University, Toyoake, Aichi, Japan

Corresponding author: Mikiko Shimizu, RN, PhD
Faculty of Nursing, Fujita Health University, School of Health Sciences, 1-98, Dengakugakubo, Kutsukake-cho, Toyoake, Aichi 470-1192, Japan
E-mail: mshimizu@fujita-hu.ac.jp

要約

【目的】

本研究では、女子大学生を対象とした継続的な経皮的ヘモグロビン値測定によるヘモグロビン値の可視化が鉄摂取量に与える影響を明らかにした。

【方法】

私立 A 大学看護学科 4 年生の女子大学生 11 名を対象に 2022 年 12 月～2023 年 2 月に調査した。経皮的ヘモグロビン値を初回／1 ヶ月後／2 ヶ月後に測定した。簡易型自記式食事歴法質問票(BDHQ)を初回／2 ヶ月後に行った。

【結果】

経皮的ヘモグロビン値の平均値±標準偏差は、初回 12.8±1.1 g/dL、1 ヶ月後 12.5±1.0 g/dL、2 ヶ月後 13.0±1.0 g/dL であった。反復測定による一元配置分散分析を行った結果、有意差はなかった。初回または 1 ヶ月後の経皮的ヘモグロビン値が平均値より低い対象はその後の測定値が増加傾向であった。初回と 2 ヶ月後の変化量の平均値±標準偏差は経皮的ヘモグロビン値 0.3±1.1 g/dL と増加した。Fe 摂取量変化量は 0.10±1.05 mg/日であり、有意差はなかった。

【結論】

経皮的ヘモグロビン値が低い学生は、初回・1 ヶ月後・2 ヶ月後の合計 3 回の継続的な測定が経皮的ヘモグロビン値の改善に効果的である可能性が示唆されたが、Fe 摂取量との関連はなかった。

キーワード

経皮的（Transcutaneous）

ヘモグロビン（Hemoglobin）

貧血（Anemia）

女子大学生（Female university students）

鉄（Iron）

本文

1．はじめに

WHO[1]の貧血実態調査では世界中の 20 億人、妊娠可能時期（15～49 歳）の女性の約 30%が貧血者である。この時期の日本人女性の貧血者は 22.5%で発展途上国と同レベルであり、その問題は深刻である。国内では第 4 次食育推進基本計画[2]において貧血が課題として挙げられている。また、近年、重要視されている女性やカップルを対象とした将来の妊娠のための健康管理を促す取組であるプレコンセプションケア[3]の 1 つとして、貧血予防が挙げられている。さらに、20 歳以上で BMI が 18.5 未満の痩せの割合は、米国では 1.7%（2017～2018 年）[4]、英国では 1.6%[5]であるのに対し、日本では 18.4%（2023 年）[6]と顕著である。BMI が 18.5 未満の痩せている人は低栄養を伴っている割合が多く、貧血の割合も多いことが報告されている[7]。このように、貧血を含む低栄養を伴っていることが多い痩せの割合が高い日本では、特に BMI18 未満を「シンデレラ体重」と定義し、問題視している。

これらのように、妊娠可能時期の日本人女性の貧血の割合は 20%以上を占めており、プレコンセプションケアや痩せの予防による貧血予防は、重要な課題の 1 つである。

貧血予防の対策として、米国ではアメリカ食品医薬品局（Food and Drug Administration）が鉄を含む強化小麦粉（Enriched flour）などの生産を法で義務づけている[8]。他国においてもサプリメントの配布など様々な国策が施行されている。しかし、日本には貧血予防に関する国策は無く、自治体や個人の努力に左右される。そこで、本研究では、いつでも、どこでも、誰にでも測定が可能で、その場ですぐに結果が分かる経皮的ヘモグロビン値測定機器に着目した。プレコンセプションケアの時期にあり、BMI が 18.5 未満の痩せの割合が多い 20～30 代[6]にある女子大学生を対象に、貧血予防策として継続的な経皮的ヘモグロビン値測定によるヘモグロビン値の可視化が女子大学生の鉄摂取量に与える影響を明らかにすることを目的とした。

2．方法

1）*研究デザイン*：縦断研究

2）*定義*：本研究では経皮的ヘモグロビン値が 12.0g/dL 未満を貧血とした。

3）*対象*：

対象は、研究の同意が得られた私立 A 大学看護学科 4 年生の女子大学生とした。

4）*調査期間*：2022 年 12 月～2023 年 2 月

5）*調査方法*：① 経皮的ヘモグロビン測定器 Rad-67 （マシモジャパン社）にて経皮的ヘモグロビン値を初回／1 ヶ月後／2 ヶ月後に測定した。各時期で 3 回測定して平均値を算出した。Rad-67 は、日本の薬事法で承認されている。専用のセンサを指先に装着すると約 1 分で経皮的ヘモグロビン値がその場で確認できる。Rad-67 は、酸化ヘモグロビン、還元ヘモグロビン、カルボキシヘ

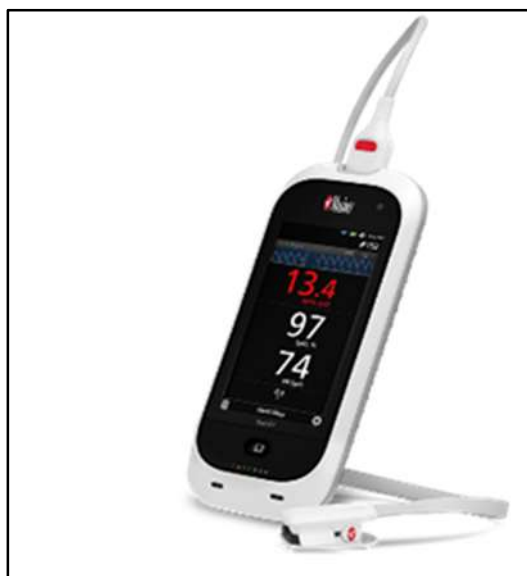

Figure 1

モグロビン、メトヘモグロビンおよび血漿を識別するために、多波長（7 波長以上）センサを使用する。信号データは、毛細血管床（指先）を経由して可視光および赤外線（範囲 500〜1,300nm）を通過し、血液の拍動周期中の吸収度の変化を測定することにより収集される。信頼性について、マシモジャパン社[9]が公開している 3,519 名を対象とした経皮的ヘモグロビン値と血液ヘモグロビン値の相関係数は r=0.91、経皮的ヘモグロビン値の標準偏差は 1.09 である。

② 簡易型自記式食事歴法質問票(Brief-type self-administered diet history questionnaire: BDHQ)を初回／2 ヶ月後に配布・回収した。質問票は、日本に住む人を対象に、通常の食事（サプリメント等を除く）から習慣的に摂取している栄養素量を、比較的簡便に、個人を対象として調べる。本研究では Fe 摂取量に使用した。調査用紙は、最近 1 ヶ月の食生活の調査であり、A3 の大きさで両面 1 枚、平均回答時間は約 15 分である。大規模な栄養疫学研究や個別保健指導に用いられることが多い[10-12]。

６）*分析方法*：

BDHQ の分析は専門業者に委託した。統計解析は SPSS 28.0 を用いた。本研究では、経皮的ヘモグロビン値の測定時期による変化を検討するために、反復測定による一元配置分散分析を実施した。従属変数は経皮的ヘモグロビン値であり、独立変数は測定時期（初回、1 ヶ月後、2 ヶ月月後）とした。初回と 2 ヶ月後の Fe 摂取量の変化を検討するために、対応のある t 検定を実施した。経皮的ヘモグロビン値と Fe 摂取量の変化量の関連を検討するため、経皮的ヘモグロビン値と Fe 摂取量の変化量の相関係数を算出した。有意水準は両側検定で 5%未満とした。

３．倫理的配慮

本研究は、藤田医科大学医学研究倫理審査委員会(承認番号:HM21-582）にて承認を得た。

４．結果

1） *対象の属性*

対象は研究の同意が得られた私立 A 大学看護学科 4 年生の女子大学生 12 名のうち、データの欠損がない 11 名（平均年齢 21.5±0.5 歳,平均 BMI 21.1±2.6）であった。

2） *経皮的ヘモグロビン値の経時的変化（Figure 2）*

経皮的ヘモグロビン値の平均値±標準偏差は、初回 12.8±1.1 g/dL、1 ヶ月後 12.5±1.0 g/dL、2 ヶ月後 13.0±1.0 g/dL であった。反復測定による一元配置分散分析を行った結果、有意差はなかった。経皮的ヘモグロビン値が初回から 2 ヶ月後に増加した 5 名のうち 3 名が初回の経皮的ヘモグロビン値が低い（貧血 2 名、12.0 g/dL：1 名）対象であった。対象 11 名の中で初回の経皮的ヘモグロビン値が低かった 4 名のうち 2 名は 1 ヶ月後の経皮的ヘモグロビン値がさらに減少したが、2 ヶ月後は増加した。

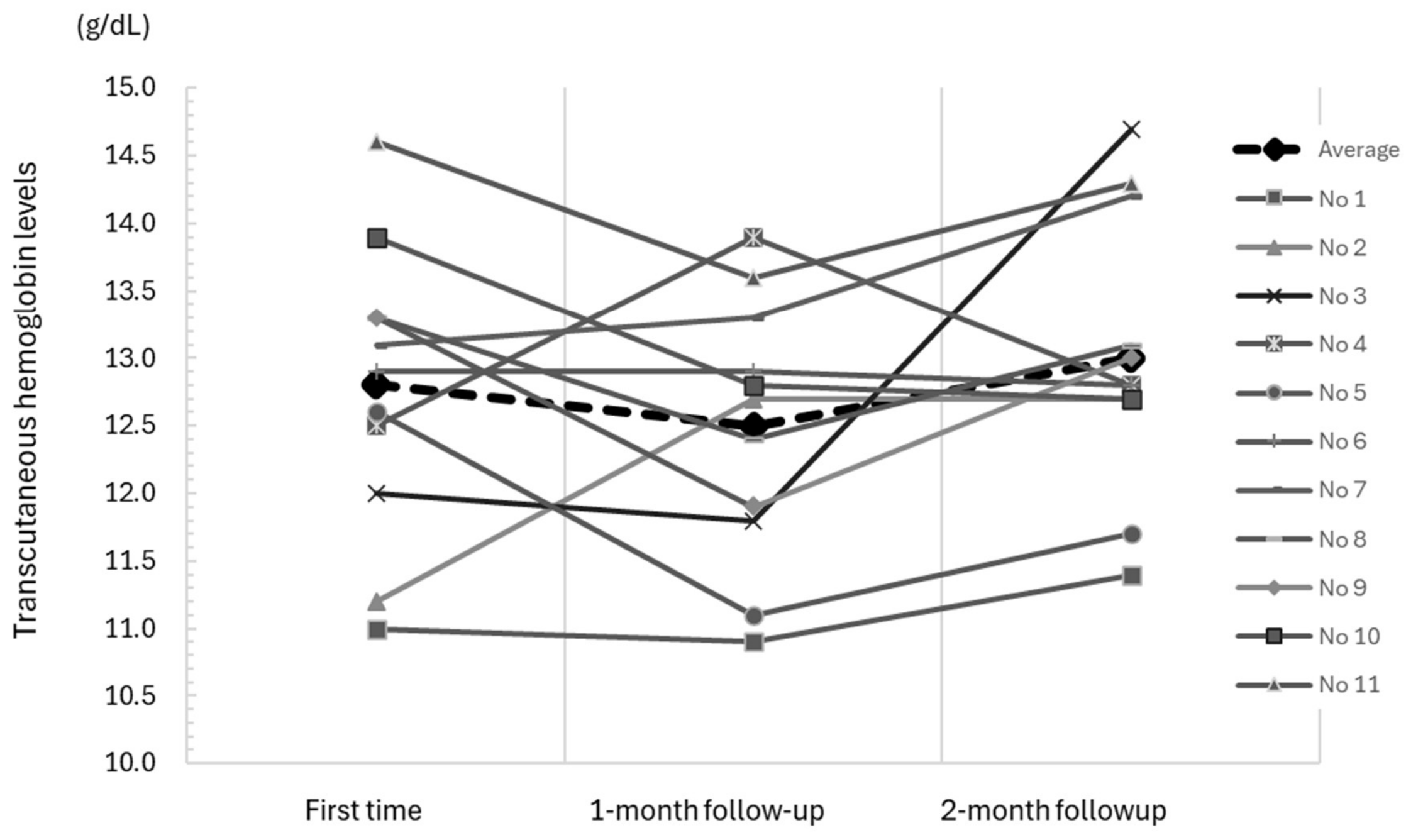

Figure 2 Change in transcutaneous hemoglobin levels from the first time to 2 months (N=11)

3）*初回と 2 ヶ月後の鉄摂取量の変化（Table 1）*

初回平均値 5.10±1.46 mg/日、2 ヶ月後平均値 5.20±1.30 mg/日であった。対応のある t 検定を行った結果、有意差はなかった。栄養補助食品を使用した対象は、初回 3 名であったが、2 ヶ月後には 5 名（初回から継続 2 名、新規使用開始 3 名）となった。初回に使用していたが 2 ヶ月後に中止したのは 1 名であった。新規使用開始の 3 名は、初回から 1 ヶ月後の経皮的ヘモグロビン値が低下した対象であった。

4）*初回と 2 ヶ月後の経皮的ヘモグロビン値と Fe 摂取量の変化量の関連(Figure 2, Table 1)*

経皮的ヘモグロビン値の変化量の平均 0.3±1.1 g/dL、Fe 摂取量の変化量の平均 0.10±1.05 mg/日であった。経皮的ヘモグロビン値と Fe 摂取量の変化量において、Spearman の順位相関係数は r=-0.036 であり、有意差はなかった。1 ヶ月後に貧血であった 4 名全員が 2 ヶ月後の経皮的ヘモグロビン値の変化量が増加した。

Table 1 Change in daily Fe intake from the first time to 2 months

	First time		2-month follow-up		
	Daily Fe intake (mg/day)	Use of supplements	Daily Fe intake (mg/day)	Use of supplements	Amount of change between the first time and 2-month follow-up
Average ± SD	5.10±1.46		5.20±1.30		0.10±1.05
No. 1	6.73	No	6.90	No	0.17
No. 2	4.74	**Yes**	4.71	**Yes**	-0.02
No. 3	4.50	No	4.11	No	-0.39
No. 4	4.43	No	5.91	No	1.48
No. 5	7.05	No	4.61	Yes	-2.44
No. 6	7.33	No	7.75	No	0.42
No. 7	4.76	No	4.89	No	0.12
No. 8	3.82	Yes	3.67	Yes	-0.15
No. 9	9.84	**Yes**	7.32	**No**	-2.52
No. 10	5.40	**No**	6.09	**Yes**	0.69
No. 11	2.46	No	3.93	Yes	1.47

＊No. 1～11：First transcutaneous hemoglobin levels in order of lowest to highest
Corresponding to the numbers in Figure 2

5．考察

本研究の新規性は、経皮的ヘモグロビン値を継続的に測定することによる貧血予防/改善の効果を検証した点である。

経皮的ヘモグロビン値の経時的変化では有意差はなかった。対象数が 11 名とサンプルサイズが小さく、データのばらつきがあったことが、有意差がなかったことに影響した可能性がある。しかし、初回と 2 ヶ月後を比較すると増加傾向であった。経皮的ヘモグロビン値が初回と比較して 2 ヶ月後に増加した対象は、初回の経皮的ヘモグロビン値が低い対象であった。1 ヶ月後の経皮的ヘモグロビン値が低かった対象は 2 ヶ月後に経皮的ヘモグロビン値が増加した。これらより、経皮的ヘモグロビン値が低い結果を受け取った対象または前回より低下した対象は、その後改善に向けて対処行動を行った可能性が示唆された。実際、初回は栄養補助食品を使用していなかった 8 名の対象のうち、2 ヶ月後には 3 名が栄養補助食品の使用を開始し、その 3 名は初回から 1 ヶ月後の経皮的ヘモグロビン値が低くなった対象であった。これより、経皮的ヘモグロビン値が低下したことが栄養補助食品の使用を開始するという対処行動に繋がった可能性が示唆された。これは、血圧や体重、スマートフォン使用時間などの数値の可視化が血圧や体重の減少、スマートフォン使用時間の減少などに効果があることが明らかとなった先行研究[13-15]と同様、経皮的ヘモグロビ

ン値測定によって数値の可視化がされたことによる効果であった可能性が示唆された。一方、BDHQ は過去 1 ヶ月の食事内容を回想する尺度であり、1 ヶ月後に再度実施すると評価対象期間が初回調査と重複するため、食生活の変化が反映されない可能性がある。そのため、本研究では食生活の変化を把握する目的で、初回と 2 ヶ月後の 2 時点で調査した。このように、本研究では栄養補助食品の使用時期を初回と 2 ヶ月後の 2 時点でのみ把握しているため、新規使用開始が 1 ヶ月後の経皮的ヘモグロビン値の低下に対する反応として行われたのか、あるいは 1 ヶ月後の測定以降に開始されたのかは特定できない。この点は本研究の限界であり、今後は栄養補助食品の使用時期をより詳細に聴取する必要がある。

初回の経皮的ヘモグロビン値が低かった対象の経時的変化は、1 ヶ月後、2 ヶ月後に徐々に増加する対象と 1 ヶ月後に減少して、2 ヶ月後に増加する対象があった。この違いより、初回の低い結果を受けて対処行動を開始した可能性のある対象と、初回と 1 ヶ月後の合計 2 回の連続して低いまたは初回から 1 ヶ月後に低下した結果を受けて対処行動を開始した可能性のある対象がいたことが示唆された。これらより、経皮的ヘモグロビン値が低かった対象が改善に向けた対処行動を促すためには、少なくとも経皮的ヘモグロビン値の測定を初回と 1 ヶ月後の 2 回は実施する必要性が示唆された。さらに、対処行動の効果を評価するために 2 ヶ月後に再測定することを設定し、少なくとも合計 3 回の経皮的ヘモグロビン値測定を行うことが経皮的ヘモグロビン値の改善に効果的である可能性が示唆された。

経皮的ヘモグロビン値の改善に向けた対処行動として、本研究では食生活の変化を指標とし、食事歴法の BDHQ を初回と 2 ヶ月後に調査した。初回と 2 ヶ月後の Fe 摂取量について対応のある t 検定を行ったが有意差はなかった。また、初回と 2 ヶ月後の経皮的ヘモグロビン値と Fe 摂取量の変化量に相関はなかった。サンプルサイズが小さいことが有意差や相関がなかったことに影響した可能性があることが課題であるが、本研究では経皮的ヘモグロビン値と Fe 摂取量に関連はなかった。BDHQ は食事歴法[16,17]であり、実際の体内での鉄吸収の程度は不明である。そのため、食べ合わせによる鉄吸収率の影響があった可能性がある。また、BDHQ では栄養補助食品の摂取頻度の質問項目はあるが、栄養補助食品の種類を問う項目はない。近年では様々な栄養補助食品が販売されており、Fe 補助食品においても Fe 含有量、種類（ヘム鉄/非ヘム鉄)、1 日の内服回数などは様々である。そのため、栄養補助食品の影響があった可能性がある。さらに、対象の月経による鉄喪失が影響した可能性がある。

6．研究の限界

本研究には 3 つの限界がある。第一にサンプル数が 11 名と少なく、統計的検出力が十分でなかった可能性がある。第二に食生活の調査において、BDHQ のみでなく、栄養補助食品の使用開始時期や種類、1 日の摂取頻度食べ合わせやなどの詳細を調査して、包括的に食生活を分析する必要があった点である。第三に月経による鉄喪失を考慮した分析が必要であった点である。

7．結論

経皮的ヘモグロビン値が低い学生において、初回・1ヶ月後・2ヶ月後の合計3回の継続的な測定は経皮的ヘモグロビン値の改善に効果的である可能性が示唆された。しかし、経皮的ヘモグロビン値の増加と Fe 摂取量との関連はなかった。そのため、Fe 吸収率や食べ合わせなどを踏まえた包括的な食生活や性周期を考慮した評価が今後の課題である。

8．利益相反

本研究における著者とマシモジャパン社との間に開示すべき利益相反はない。その他の著者と企業などとの間においても開示すべき利益相反はない。

9．謝辞

この研究に参加いただいたすべての方に感謝申し上げます。尚、本研究は科学研究費補助金基盤研究(C)（課題番号：24K13873）の助成を受けて実施した。

引用文献

1. WHO. Anaemia; 2025.<https://www.who.int/health-topics/anaemia#tab=tab_1>(Accessed July 9, 2025)
2. Ministry of Agriculture, Forestry and Fisheries. The Fourth Basic Plan for the Promotion of Shokuiku;2021 (in Japanese). <https://www.maff.go.jp/j/syokuiku/wpaper/r2/r2_h/book/part1/chap3/b1_c3_1_02.html> (Accessed July 9, 2025)
3. WHO. Preconception care: Maximizing the gains for maternal and child health - Policy brief; 2013.<https://www.who.int/publications/i/item/WHO-FWC-MCA-13.02>(Accessed July 9, 2025)
4. Cheryl D. Fryar, M.S.P.H., Margaret D. Carroll, M.S.P.H., and Joseph Afful, M.S. Products - Health E Stats - Prevalence of Underweight Among Adults Aged 20 and Over: United States, 1960–1962 Through 2017–2018;2021. <https://www.cdc.gov/nchs/data/hestat/obesity-adult-17-18/obesity-adult.htm>(Accessed July 9, 2025)
5. Nicholson BD, Aveyard P, Bankhead CR, Hamilton W, Hobbs FDR, Lay-Flurrie S. Determinants and extent of weight recording in UK primary care: an analysis of 5 million adults' electronic health records from 2000 to 2017. BMC Med. 2019 Nov 29;17(1):222. doi: 10.1186/s12916-019-1446-y. PMID: 31783757; PMCID: PMC6883613.
6. Ministry of Health, Labor and Welfare. Summary of the 2023 National Health and Nutrition Examination Survey;2024(In Japanese). <https://www.cdc.gov/nchs/nhanes/index.html>(Accessed July 9, 2025)
7. Iizuka K, Sato H, Kobae K, Yanagi K, Yamada Y, Ushiroda C, Hirano K, Ichimaru S, Seino Y,

Ito A, Suzuki A, Young N: Japanese Underweight Women with “Cinderella Weight” Are Prone to Malnutrition, including Vitamin Deficiencies, Nutrients, 2023, 15(9), 2216. doi:10.3390/nu15092216. PMID: 37409654; PMCID: PMC10181057.

8. Food and Drug Administration. Guidance for Industry: Questions and Answers on FDA’s Fortification Policy;2015.
＜https：//www.fda.gov/regulatory－information/search－fda-guidance-documents/guidance-industry-questions-and-answers-fdas-fortification-policy ＞(Accessed July 9, 2025)
9. MasimoCo. Rad-67™ Pulse CO Oximeter for Spot Check;2025 (in Japanese).
<https://masimo.co.jp/rad-67/index.htm>(Accessed July 9, 2025)
10. Sasaki S, Ushio F, Amano K, Morihara M, Todoriki O, Uehara Y, Toyooka E.　Serum Biomarker-based Validation of a Self-administered Diet History Questionnaire for Japanese Subjects. J Nutr Sci Vitaminol　2000;46:285-96.
11. Sasaki S, Yanagibori R, Amano K. Self-Administered Diet History Questionnaire Developed for Health Education: A Relative Validation of The Test-Version by Comparison with 3-Day Diet Record in Women. J Epidemiol　1998;8:203-15.
12. Sasaki S, Yanagibori R, Amano K. Validity of a Self-Administered Diet History Questionnaire for Assessment of Sodium and Potassium. Jpn Circ J　1998;62:431-5.
13. Tagi M, Tamaki Y, Morikawa T, Matsuhisa M, Moriguchi H. The Behavior Modification to the Lifestyle by Healthy Condition Visualization which Utilized NFC Communication Pedometer. Japan Journal of Medical Informatics 2014; 34: 281-91(in Japanese).
14. Nishiguchi Y, Yoneyama N. Effects of Goal Setting and Graphic Feedback on the Amount of Time College Students Used Their Smartphones and Their Sleep Quality. Japanese Journal of Behavior Analysis 2024; 38: 110–9(in Japanese).
15. Yokoyama J, Nagai M, Yamamoto M, Discussion about Visualization of Lifestyle. Annual Conference of Japan Management Diagnosis Association 2009;91-4(in Japanese).
16. Kobayashi S, Murakami K, Sasaki S, Okubo H, Hirota N, Notsu A, Fukui M, Date C. Comparison of relative validity of food group intakes estimated by comprehensive and brief-type self-administered diet history questionnaires against 16 d dietary records in Japanese adults. Public Health Nutr 2011; 14: 1200-11.
17. Kobayashi S, Honda S, Murakami K, Sasaki S, Okubo H, Hirota N, Notsu A, Fukui M, Date C. Both comprehensive and brief self-administered diet history questionnaires satisfactorily rank nutrient intakes in Japanese adults. J Epidemiol 2012; 22: 151-9
